# Clinical Profiles and Therapeutic Interventions in Occult Paraquat Poisoning: A Comparative Report of Two Cases

**DOI:** 10.1002/ccr3.72865

**Published:** 2026-06-07

**Authors:** Minhong Zhao, Shuai Zhou, Ying Zhang, Eryan Yuan, Min Peng, Ying Gao

**Affiliations:** ^1^ Tianjin Medical University Tianjin China; ^2^ Department of Critical Care Unit Tianjin Medical University General Hospital Tianjin China

**Keywords:** acute respiratory distress syndrome, diagnosis and management, different exposure routes, inhalation toxicity, multiple organ dysfunction syndrome, paraquat poisoning

## Abstract

Delayed diagnosis invariably worsens outcomes in occult paraquat poisoning regardless of exposure route. Implement early urinary screening for suspected cases. Tailor antioxidant and anti‐fibrotic timing to toxicokinetic patterns to mitigate irreversible pulmonary damage.

## 1 Introduction

The case fatality rate for moderate to severe acute paraquat ingestions remains extremely high. Current clinical data estimate that this mortality consistently spans from 60% to 90% in symptomatic patients [1]. Furthermore, no specific or effective treatment antidote is currently available to reverse this toxicity [2]. As a highly effective bipyridine contact herbicide, paraquat exerts its toxic effects primarily through the induction of oxidative stress and the formation of irreversible interstitial lung fibrosis. Although lung injury is a hallmark feature, paraquat can affect multiple organs, leading to MODS [3]. Notably, the increasing prevalence of atypical exposure patterns, combined with early nonspecific manifestations such as gastrointestinal and respiratory symptoms, often results in a high misdiagnosis rate during the initial evaluation of occult poisoning. In this context, we retrospectively analyzed two cases of insidious subacute paraquat poisoning admitted to our department in 2024. We further investigated the toxicokinetic evolution, dynamic imaging findings, and optimal timing for initiating extracorporeal life‐support interventions, aiming to provide an evidence‐based foundation for early clinical identification and comprehensive management of paraquat poisoning. A clinical decision flowchart was concurrently developed to facilitate rapid identification of such patients (Figure 1).

**FIGURE 1:**
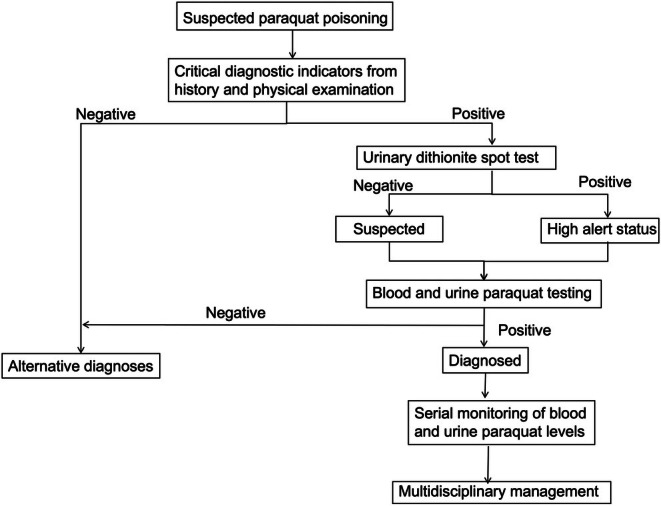
Clinical decision flowchart.

## 2 Case Presentation

### 2.1 Case 1

#### 2.1.1 Case History/Examination

A 22‐year‐old male presented to the emergency department with a 6‐day history of progressive chest pain and dyspnea, leading to admission on February 10, 2024. Four days prior to this admission, the patient had sought medical attention, but he declined hospitalization despite initial imaging findings. Upon readmission, his clinical condition had deteriorated rapidly. Physical examination revealed severe respiratory distress and extensive subcutaneous crepitus across the neck and chest.

#### 2.1.2 Differential Diagnosis

The initial presentation of spontaneous pneumomediastinum, cervical emphysema, and rapid respiratory decline in a healthy young male raised a broad differential diagnosis. This included severe viral or atypical pneumonia, acute respiratory distress syndrome (ARDS) secondary to occult infection, ruptured esophagus (Boerhaave syndrome), and toxic inhalation injury. Although the patient and his family initially denied any history of toxicant exposure, the atypical progression and severe multi‐organ involvement strongly suggested occult poisoning, which remained a primary suspicion.

#### 2.1.3 Investigations

An initial computed tomography (CT) scan obtained 4 days prior to admission revealed prominent cervical and mediastinal emphysema accompanied by pulmonary interstitial emphysema (Figure 2A,B). Upon readmission, repeat imaging demonstrated rapidly worsening diffuse pulmonary infiltrates and respiratory distress (Figure 2C,D). Laboratory findings indicated systemic inflammation (WBC 28.7 × 10^9^/L, CRP 11.7 mg/dL) and multi‐organ injury (Cr 189 μmol/L, ALT 204 U/L). He progressed to severe ARDS within 48 h (PaO_2_/FiO_2_ ratio < 100 mmHg, radiographic involvement > 85%), failing lung‐protective ventilation. Although an initial urine paraquat test yielded a negative result, a subsequent targeted investigation of the patient's personal items revealed a video documenting inhalation of an unknown substance. Analysis of the residual solution from his nebulization device confirmed the presence of methyl viologen, the active component of paraquat, establishing the diagnosis of inhaled paraquat poisoning via nebulization.

**FIGURE 2:**
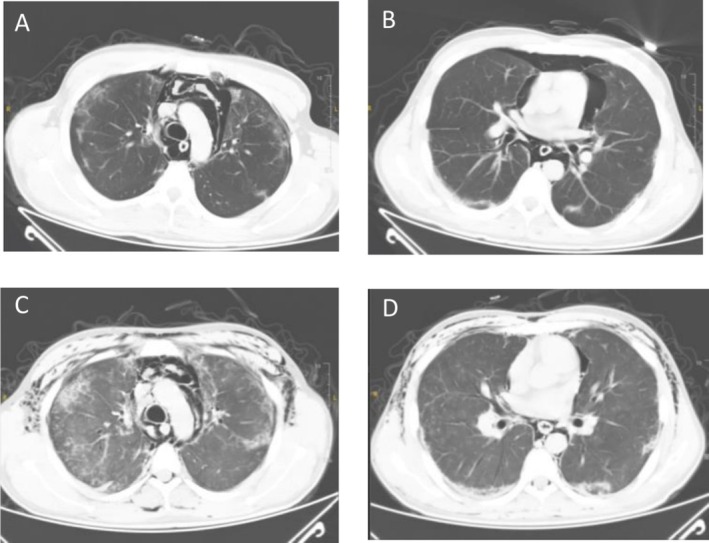
Patient's chest CT. (A, B) The chest CT obtained on February 6 revealed bilateral multifocal patchy opacities and linear densities, with concomitant gaseous infiltration evident within the soft tissue planes, mediastinum, peribronchovascular spaces of both lungs, and interlobar fissures. (C, D) The chest CT performed on February 10 (at admission) demonstrated interval progression of bilateral pulmonary patchy opacities, with notable worsening of pneumomediastinum and subcutaneous emphysema compared to prior imaging.

#### 2.1.4 Treatment

Due to the failure of lung‐protective mechanical ventilation, veno‐venous extracorporeal membrane oxygenation (V‐V ECMO) was emergently initiated. Following diagnostic confirmation, a multimodal therapeutic strategy was implemented: (1) targeted detoxification: methylprednisolone pulse therapy to inhibit alveolar injury, cevilastat sodium to block neutrophil oxidative stress, and aerosolized N‐acetylcysteine combined with high‐dose vitamin C/E to scavenge free radicals; (2) multi‐organ support: ECMO combined with ultra‐lung protective ventilation (plateau pressure < 25 cmH_2_O, driving pressure < 10 cmH_2_O); combined with CRRT for toxin removal, hepatoprotection, renal protection, and nutritional support.

#### 2.1.5 Outcome and Follow‐Up

After treatment, the patient's oxygen saturation improved (PaO_2_/FiO_2_ > 300 mmHg), chest radiographs showed > 50% absorption of exudates in both lungs (Figure 3), ventilator parameters were gradually weaned, and related laboratory indices returned to normal (Figure 4). However, the patient refused to continue therapy after regaining full consciousness and requested a voluntary discharge against medical advice. Following discharge, no further laboratory tests or radiologic evaluations were performed. The patient died at home 3 days later. This rapid development of fatal progressive respiratory failure was deduced based on the severe clinical status of the patient at the time of discharge and the established toxicological mechanisms of paraquat‐induced lung injury.

**FIGURE 3:**
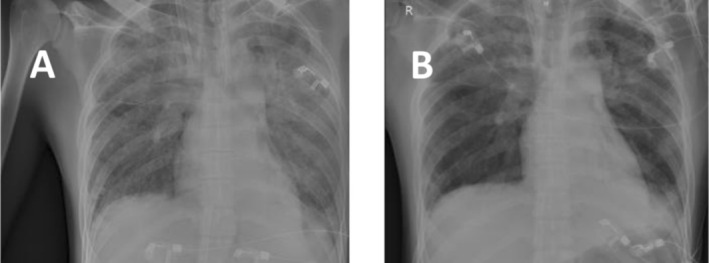
Chest X‐ray. (A) On February 16 (6th day in hospital), the patient's condition deteriorated further. Chest radiograph revealed progression of bilateral lung involvement, including increased lung markings, diffuse haziness, and enlarged patchy opacities compared to prior imaging. Additionally, blunting of the right costophrenic angle and obscuration of the right hemidiaphragm were noted, consistent with radiographic progression of pulmonary pathology. (B) On February 21 (the 12th day in hospital), after treatment, the extent of the patient's multiple patchy shadows in both lungs on chest radiographs was reduced and the right rib diaphragm angle as well as the diaphragm were clearer than before.

**FIGURE 4:**
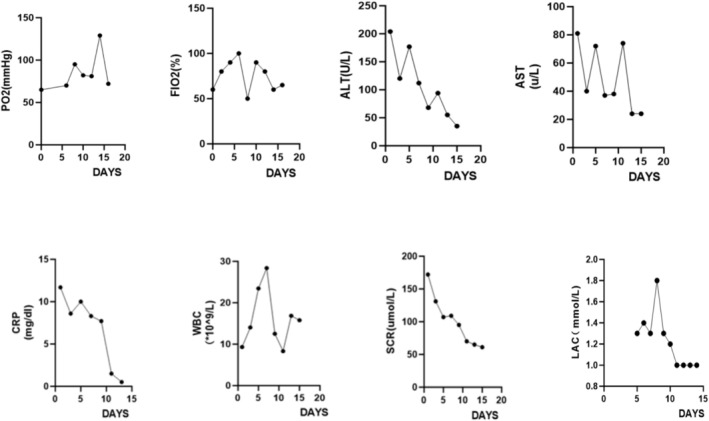
Trends in laboratory changes. The figure shows the changes in the patient's infection index, liver and kidney function, and respiratory‐related index. After comprehensive treatment, the overall trend of the patient is getting better.

### 2.2 Case 2

#### 2.2.1 Case History/Examination

A 42‐year‐old male with a history of hypertension and diabetes was admitted to the ICU on December 8, presenting with a 4‐day history of vomiting and diarrhea, and a 2‐day history of dyspnea and oliguria. On examination, he was febrile (37.7°C) with oxygen desaturation (SpO_2_ 79%) and severe jaundice.

#### 2.2.2 Differential Diagnosis

The clinical triad of severe acute hypoxemic respiratory failure, acute kidney injury, and profound hepatic jaundice suggested a fulminant systemic insult. The differential diagnosis included severe sepsis or septic shock with multiple organ dysfunction syndrome (MODS), leptospirosis, acute hemolytic anemia, and fulminant toxic exposure. The rapid evolution toward severe pulmonary fibrosis and multi‐organ failure raised a high index of suspicion for acute poisoning.

#### 2.2.3 Investigations

The chest CT revealed diffuse ground‐glass opacities (Figure 5). Laboratory findings showed acute kidney injury (Cr 1011 μmol/L), hyperbilirubinemia (TBil 203 μmol/L), and hypoxemia (PaO_2_ 65 mmHg). Upon repeated history‐taking and a detailed review of the patient's personal belongings, a video documenting suicidal intent was discovered. Blood specimens were immediately dispatched for toxicological screening, which confirmed a positive serum paraquat level, establishing the diagnosis of acute paraquat poisoning.

**FIGURE 5:**
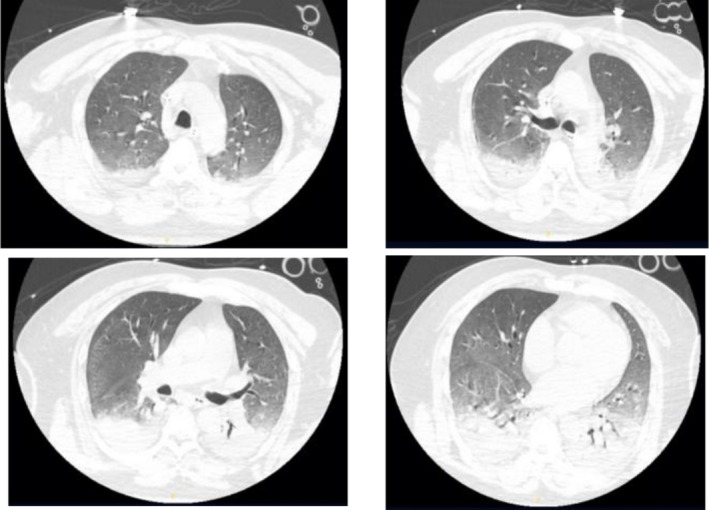
Patient's chest CT. Diffuse multiple patchy ground‐glass density shadows and patchy shadows in both lungs; solid shadows were seen in the lower lobes of both lungs, and inflated bronchial signs were seen.

#### 2.2.4 Treatment

Endotracheal intubation with mechanical ventilation and continuous renal replacement therapy (CRRT) was immediately initiated. Following toxicological confirmation, a comprehensive multidisciplinary regimen was deployed: (1) Toxin removal and organ support: CRRT combined with hemoperfusion to remove circulating toxins; V‐V ECMO combined with endotracheal intubation connected with ventilator‐assisted ventilation to improve oxygenation; (2) immunosuppressive and antioxidant therapies were adjusted based on patient organ function. Intravenous methylprednisolone was administered at 500 mg daily for three consecutive days. Cyclophosphamide was temporarily withheld due to early hepatic and renal impairment but was safely initiated at 0.8 g daily on the third day after blood purification improved organ function. Antioxidants included intravenous reduced glutathione at 2400 mg daily, intravenous vitamin C at 2 g every 12 h, and gastric N‐acetylcysteine at 0.6 g every 12 h; (3) Organ function maintenance and mucosal protection included intravenous octreotide at 0.6 g daily, ademetionine for cholestasis, sodium creatine phosphate for myocardial support, and lactulose for intestinal regulation. Kangfuxin solution was administered at 10 mL three times daily via a gastric tube to accelerate mucosal healing; (4) anti‐infection management utilized empirical broad spectrum antimicrobial therapy. Piperacillin sodium tazobactam was administered intravenously to cover potential secondary infections from gram negative and anaerobic pathogens.

#### 2.2.5 Outcome and Follow‐Up

As a result of these interventions, the oxygenation index rose to 250 mmHg on ECMO support. On review of the chest X‐ray, the patchy shadow was partially absorbed compared to the previous one (Figure 6), and the patient's hepatic and renal functions were partially restored (Figure 7). However, lung transplantation was abandoned due to financial constraints experienced by the family, resulting in the voluntary discharge of the patient against medical advice. Following this discharge, no further laboratory tests or radiologic evaluations were performed to monitor the condition. The patient died at home 1 week later. This terminal outcome of fatal respiratory failure combined with progressive multi‐organ failure was deduced based on the clinical status of the patient at the time of discharge and the established toxicological mechanisms of paraquat‐induced tissue destruction.

**FIGURE 6:**
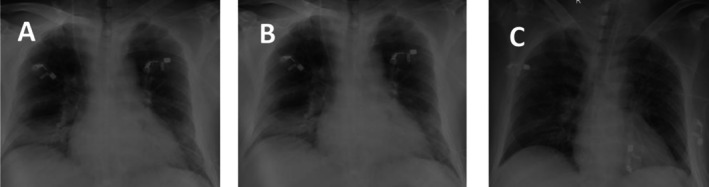
Chest X‐ray. (A) On December 8, chest radiography revealed diffuse decreased lung radiolucency with extensive consolidations predominantly in bilateral lower lobes, accompanied by cardiomegaly and blunted costophrenic angles. (B) On December 11, imaging demonstrated progression of left pulmonary pathology, with enlarged extent and increased attenuation of multifocal consolidations. (C) On December 12, post‐therapeutic imaging revealed modest improvement in right lung radiolucency and marked restoration of left lung transparency with significant reduction in consolidations.

**FIGURE 7:**
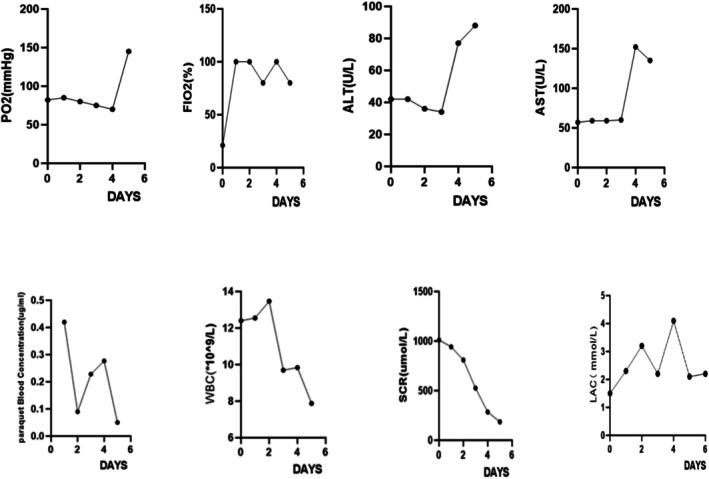
Trends in laboratory changes. After comprehensive treatment, the patient's hepatic and renal function, oxygen partial pressure, and oxygen concentration recovered from the previous period; the concentration of paraquat in the blood, after CRRT combined with hemoperfusion treatment, the overall trend decreased. Notably, because the patient received continuous renal replacement therapy immediately upon intensive care unit admission, the systemic blood lactate levels remained relatively stable with minimal fluctuations throughout the clinical course.

## 3 Discussion

Paraquat is a highly effective nonselective contact herbicide, and covert paraquat intoxication poses significant diagnostic challenges.

At the level of toxicological mechanisms, its characterization as mediating organ damage through multiple pathways has been widely confirmed.

Its bipyridyl salt structure undergoes continuous intracellular redox cycling catalyzed by NADPH oxidase in vivo. This process generates excessive superoxide anions and triggers an extensive oxidative stress cascade reaction [4]. The resulting systemic free radical damage leads to rapid lipid peroxidation [5], progressive mitochondrial injury through the depolarization of mitochondrial membrane potential [6], and severe DNA damage [7]. This widespread subcellular disruption ultimately explains the development of profound multi‐organ dysfunction.

Different routes of exposure directly alter these toxicokinetic characteristics and tissue distribution patterns. During aerosolized inhalation, the aerodynamic deposition of toxic fine particles allows them to reach the terminal bronchioles and alveolar lumen directly. Alveolar surfactants enhance toxin solubility, which facilitates rapid absorption through epithelial active transport mechanisms directly into the pulmonary capillary bloodstream. This localized deposition causes immediate alveolar epithelial cell destruction and massive pulmonary accumulation while bypassing hepatic first pass metabolism. Conversely, the oral route relies on gastrointestinal absorption via specific organic cation transporter [8]. This alternative pathway leads to initial toxin distribution in the kidneys and liver, followed by a more gradual, active accumulation in lung tissue over subsequent days.

Clinically, the definitive diagnosis of paraquat poisoning usually relies on a clear history of poisoning and typical clinical manifestations. The prognosis is closely related to the poisoned dose, the timing of treatment, and the individual differences of patients. Currently, there is no specific antidote; comprehensive treatment is the key [9], but the prognosis is poor, especially if the diagnosis and treatment are delayed; the patient often dies due to multi‐organ failure.

In the two cases, the patients were admitted to the hospital with no clear history of paraquat exposure and presented only with chest pain, mediastinal emphysema, vomiting and diarrhea, which were initially diagnosed as mediastinal emphysema and diarrhea. However, when conventional treatment was ineffective and the condition worsened, the possibility of paraquat poisoning was promptly considered and the diagnosis was confirmed by toxicologic testing and history tracing. This suggests that in our clinical work, we should be highly alert to the possibility of insidious paraquat poisoning in patients with unexplained respiratory symptoms, especially those accompanied by lung lesions and systemic multi‐organ lesions. Although both cases were paraquat poisoning, there were significant differences in clinical manifestations and disease progression due to different routes of exposure. By comparing different poisoning routes, significant differences in toxicokinetics, organ damage characteristics, and prognostic mechanisms were revealed (Table 1).

**TABLE 1:** Comparison of different poisoning routes.

	Case 1 (poisoning by nebulization)	Case 2 (poisoning by oral route)
Poisoning mechanism	Direct aerodynamic deposition in lower respiratory tract bypassing hepatic first pass metabolism; rapid systemic absorption via alveolar epithelial capillary membrane with early pulmonary concentration	Gastrointestinal absorption via organic cation transporters; systemic distribution through bloodstream targeting kidneys and liver before accumulating in lung tissue
Clinical manifestation	Early pharyngeal pain, hoarseness, cough, dyspnea, rapidly progressing to severe lung lesions such as pulmonary edema and mediastinal emphysema	Early pharyngeal pain, hoarseness, cough, dyspnea, rapidly progressing to severe lung lesions such as pulmonary edema and mediastinal emphysema
Treatment measures	Removal from toxic environment, high‐flow oxygen/mechanical ventilation; nebulized glucocorticoids; blood purification; antioxidant therapy	Gastric lavage, catheterization to remove toxins; hemoperfusion/dialysis; high doses of antioxidants, immunosuppressants
Prognosis	Early intervention partially relieves lung lesions, but risk of pulmonary fibrosis is high; mortality is higher	Poor prognosis, multiple organ failure in high dose ingestion; survivors often left with severe pulmonary fibrosis

In Case 1, the nebulized particles were directly deposited in the respiratory tract and alveoli, and were rapidly absorbed by the alveolar epithelial cells into the bloodstream, which led to the first manifestation of acute respiratory distress syndrome, and the characteristic three‐stage evolution of “hairy glass shadow‐solid lesion‐interstitial fibrosis” was shown on chest CT, which was in line with the pathological process of direct damage to the alveolar epithelial cells. Case 2 showed a classic three‐stage course: initial stage (0–3 days) with digestive corrosion damage, progressive stage (4–14 days) with hepatic and renal failure, and terminal stage (> 14 days) with respiratory failure due to pulmonary fibrosis. The pathological basis for this fulminant fibrosis in Case 2 lies in the severe, irreversible destruction of alveolar epithelial cells driven by high systemic toxin accumulation. This widespread epithelial necrosis breaches the structural integrity of the basement membrane and triggers an uncontrolled, destructive fibroproliferative response characterized by the rapid formation of fibroblastic foci and excessive, disorganized extracellular matrix deposition within the alveolar architecture. Of note, the rare complication of mediastinal emphysema observed in Case 1 may be related to paraquat‐induced alveolar basement membrane injury resulting in alveolar rupture.

In terms of therapeutic strategies, the multimodal combined regimen recommended by current guidelines showed limitations in this group of cases. Although both patients received ECMO support, mechanical ventilation parameter settings need to be carefully weighed: high‐concentration oxygen therapy may exacerbate oxidative stress injury, whereas hypoxic strategies, while delaying ROS generation, may exacerbate tissue hypoxia. Both patients received ECMO adjuvant therapy, but it can only be used as a transitional support waiting for a suitable lung source to provide temporary oxygenation support and alleviate complications caused by respiratory failure [10]. It does not by itself block or inhibit the progression of paraquat‐induced lung lesions. This view is further validated by the recurrent condition and eventual death of two patients after withdrawal of ECMO. Notably, Case 2 used CRRT combined with resin hemoperfusion, which resulted in paraquat clearance of 65%–78%, but the therapeutic time window has a decisive impact on the prognosis [11]. In our case, both patients missed the optimal clearance period, which may be a key factor for treatment failure.

We are also reminded that for unexplained multi‐organ failure with rapidly progressive pulmonary fibrosis, even if the history of toxic exposure is negative, we should establish a rapid screening process for insidious poisoning. We should establish a rapid screening process for insidious poisoning. Incorporating a rapid urinary paraquat test using the colloidal gold assay into the routine workup of unexplained multi‐organ failure is highly practical. This qualitative screening test offers excellent diagnostic specificity but possesses a critical false negative rate in delayed presentations due to the rapid renal clearance of the compound within 24 h. Despite this physiological limitation, its low operational cost, rapid turnaround time of under 15 min, and minimal laboratory infrastructure requirements make it an exceptionally cost effective tool for emergency triage, preventing catastrophic diagnostic delays in occult poisonings. Both cases were treated with multidisciplinary comprehensive therapy, but with different focuses: the nebulized inhalation poisoner was treated with intensive airway management to remove local residual toxins and combined with restrictive oxygen therapy to delay oxidative stress injury; the oral poisoner was treated with CRRT combined with hemoperfusion to prioritize the removal of circulating toxins, and targeted support for hepatic and renal function impairment was also provided. Paraquat activates related pathways through ROS generation, promoting fibroblast proliferation and collagen deposition [12]. Despite high‐dose antioxidant and immunosuppressive therapies in both patients, the interventions failed to halt fibrotic progression. These findings suggest that novel therapeutic strategies targeting reactive oxygen species associated pathways represent a critical direction for future investigation. Recent preclinical studies from 2024 through 2026 focus on specific small molecule interventions to disrupt these cascades. For instance, the pharmacological activation of the nuclear factor erythroid 2 related factor 2 pathway using candidate molecules like omaveloxolone or oltipraz has demonstrated efficacy in restoring intracellular glutathione pools and reducing lung injury in animal models. Similarly, the targeted inhibition of NADPH oxidase using the selective inhibitor setanaxib represents a major breakthrough, as it directly suppresses the intracellular redox cycling required for paraquat to generate superoxide anions. Beyond small molecules, preclinical gene therapy strategies utilizing advanced delivery vehicles are under active development. Recent protocols leverage lipid nanoparticles to transport small interfering RNA designed to silence transforming growth factor beta one signaling, effectively halting the downstream activation of myofibroblasts and subsequent extracellular matrix accumulation in lung tissues. Transitioning these molecular and genetic approaches into clinical translation remains an essential challenge for future toxicological research.

## 4 Conclusion

In conclusion, this report provides significant clinical insights into occult paraquat poisoning via different routes. Clinical management necessitates an integrated precision treatment strategy addressing exposure route, toxicokinetics, and target organ protection. Furthermore, these cases underscore the critical need for enhanced regulation of paraquat to prevent its misuse or accidental exposure, which is essential for preventing such poisonings. Future efforts should focus on researching and establishing evidence‐based guidelines to standardize management protocols, while simultaneously exploring novel therapeutic approaches to improve outcomes and reduce mortality.

## Author Contributions

**Minhong Zhao:** conceptualization, data curation, methodology, resources, writing – original draft. **Shuai Zhou:** conceptualization, formal analysis, methodology, writing – original draft. **Ying Zhang:** data curation, methodology. **Eryan Yuan:** investigation, methodology. **Min Peng:** writing – review and editing. **Ying Gao:** writing – review and editing.

## Funding

The authors have nothing to report.

## Ethics Statement

The study was approved by the TianJin Medical University General Hospital Ethics Committee.

## Consent

Written informed consent was obtained from the patients to publish this report in accordance with the journal's consent policy.

## Conflicts of Interest

The authors declare no conflicts of interest.

## Data Availability

The data that support the findings of this study are available from the corresponding author upon reasonable request.
